# Editorial: Reviews in ubiquitin signaling: 2022

**DOI:** 10.3389/fmolb.2023.1275393

**Published:** 2023-08-23

**Authors:** Adam J. Fletcher, Peter D. Mabbitt

**Affiliations:** ^1^ MRC-University of Glasgow Centre for Virus Research, School of Infection and Immunity, University of Glasgow, Glasgow, United Kingdom; ^2^ Scion, Rotorua, New Zealand

**Keywords:** ubiquitin (Ub), small ubiquitin-like modifier (SUMO), deubiquitinating enzyme (DUB), non-lysine ubiquitination, ubiquitin E3 ligase

This collection of reviews highlights the central role that ubiquitin, and ubiquitin-like proteins, play in biology and how hypothesis driven use of new and existing biochemical tools continues to expand our understanding of cellular physiology. We begin with two reviews that focus on proteostasis in specific disease states. Sap et al. review the role of the ubiquitin-proteasome system (UPS) in Huntington’s disease, suggesting that defects in Huntingtin (HTT)-UPS targeting might be alleviated through understanding differential ubiquitination of “normal” *versus* polyQ-expanded mutant HTT. Zhu et al. review small ubiquitin-like modifiers (SUMO) signalling in cancer stem cells. Small molecules that alter flux through SUMOylation cascades and influence cancer cell metastatic properties point towards potential therapeutic targets. These reviews point to the need for specific inhibitors of ubiquitinating and deubiquitinating enzymes (DUBs) as well as new protein-targeting chimeras (PROTACs) to direct the degradation of misregulated or pathogenic proteins.

There is a considerable gap between clinically useful drugs and tool-compounds for the ubiquitin system. Remarkable progress has been made in both areas. Kennedy et al. discuss recent developments in fragment-based drug discovery for the Ubiquitin system in their mini-review. Typically, covalent fragments target the catalytic cysteines of Ubiquitin E2 conjugating enzymes (E2s), cysteine dependent E3 ubiquitin ligases (cys-E3s) and DUBs. Kennedy and colleagues suggest that fragments which target non-cysteine nucleophiles may be the basis for the next-generation of tool compounds.

De Cesare provides a timely personal perspective on dissecting the Ubiquitin system with matrix assisted laser desorption/ionization time-of-flight (MALDI-TOF) mass spectrometry (MS). She and others have put this technique to use in identifying DUBs and E2s with non-lysine activity (Wang et al., 2009; De Cesare et al., 2021; Rehman et al., 2023). Key questions remain around the physiological roles of non-lysine ubiquitination. Given the context dependent and promiscuous nature of E2, E3 and DUB activity *in vitro* experiments are likely to reveal more surprising substrates. We anticipate that new biochemical tools will be required to validate these observations *in vivo*.

Continuing this theme, Kelsall provides an in-depth review of ubiquitination beyond lysine. Transfer of ubiquitin to serine, threonine, and non-proteinaceous substrates has re-entered the spotlight in recent years. Bacterial effector proteins in the SidE family bypass the ubiquitination cascade completely to link ubiquitin to serine via a phosphoribosyl linkage (Tomaskovic et al., 2022). In eukaryotes specialised cys-E3 domains with non-lysine reactivity reside within large proteins (Pao et al., 2018; Kelsall et al., 2019; Ahel et al., 2020; Mabbitt et al., 2020; Ahel et al., 2021; Otten et al., 2021). A caveat to this is that RING-between-RING (RBR) E3s, such as RNF216, which catalyse ubiquitin transfer to lysine have ubiquitin esterification activity *in vitro* (Wang et al., 2023). The presence of, at the minimum, a cysteine-histidine catalytic dyad appears to be sufficient for Ub transfer to ester substrates *in vitro* (Pao et al., 2018; Wang et al., 2023). Given that catalytic triads are common and have evolved multiple time independently, it is conceivable that there are many more enzymes which transfer ubiquitin to ester substrates (Dodson and Wlodawer, 1998; Squair and Virdee, 2022). Ubiquitination of non-proteinaceous targets vastly expands the already enormous influence of these small globular proteins on cell biology. With new chemical probes and sensitive “omics” techniques coming online, it is an exciting time to be a ubiquitin biologist.
